# Physician Opioid Prescribing Attitudes in Michigan: An Exploratory Pilot Study of Age-Gender Interactions and the Awareness-Uncertainty Paradox

**DOI:** 10.7759/cureus.96587

**Published:** 2025-11-11

**Authors:** Deepti Sanku, Kush Patel, Aiman Almasnaah, Caleb Zimmerman, Patrick Fakhoury, Aditya Shah, Daniel Clauw, Peter Dijkstra

**Affiliations:** 1 College of Medicine, Central Michigan University, Mt. Pleasant, USA; 2 Chronic Pain and Fatigue Research Center, University of Michigan, Ann Arbor, USA; 3 College of Science and Engineering, Central Michigan University, Mt. Pleasant, USA

**Keywords:** attitude of health personnel, demographic factors, michigan, opioid-related disorders, pain management

## Abstract

Background

Physician attitudes toward opioid prescribing directly influence patient access to pain management while potentially contributing to misuse. This exploratory pilot study examined the demographic predictors of physician opioid prescribing attitudes in Michigan and explored relationships between risk awareness and prescribing difficulty.

Methods

This cross-sectional exploratory survey assessed opioid-prescribing attitudes among 51 practicing physicians in Michigan. Prescribing caution was measured using three Likert scale items, with composite scores analyzed using t-tests, analysis of variance (ANOVA), and multiple linear regression to test demographic predictors and interactions.

Results

No significant main effects emerged for rural versus urban practice (P=0.906), gender (P=0.554), or specialty type (P=0.532). However, a potential age×gender interaction was observed (β=0.330±0.15, P=0.033), suggesting that age effects on prescribing attitudes may differ between male and female physicians. Physicians recognizing opioid misuse as a patient problem tended to report greater prescribing difficulty (r=0.611, P<0.001, 95% CI (0.41, 0.76)). Condition-specific endorsement revealed patterns favoring cancer pain (50/51, 98.0%) and sickle cell disease (40/51, 78.4%), with lower endorsement for headaches (4/51, 7.8%) and fibromyalgia (2/51, 3.9%).

Conclusions

This preliminary study suggests that awareness of opioid risks may be associated with increased clinical uncertainty. Age and gender may interact in complex ways that warrant further investigation in larger samples. Future research should explore whether educational interventions addressing both risk awareness and decision support could reduce prescribing uncertainty.

## Introduction

The opioid crisis remains one of the most significant public health challenges facing the United States [1], accounting for 76% of all drug overdose deaths nationally in 2023. In Michigan, the situation is even more severe, with opioid overdose deaths comprising 80% of all drug overdose fatalities (2,305 out of 2,882 deaths) in that same year [2]. This makes understanding of prescribing patterns within the state critically important.

Physician attitudes toward opioid prescribing play a pivotal role in the crisis, creating a dual responsibility dilemma: clinicians must ensure adequate pain management while simultaneously preventing misuse and diversion [3,4]. This tension places physicians at the intersection of competing ethical and clinical imperatives. Research suggests that prescribing behaviors are influenced by complex factors including demographic characteristics, training experiences, and practice contexts [5,6], yet the specific nature of these relationships remains poorly understood.

Demographic predictors of prescribing attitudes

Prior research has identified several demographic factors that may shape opioid prescribing patterns. Geographic setting appears relevant, as rural physicians face distinct challenges, including limited access to alternative pain management resources and higher rates of opioid misuse in their patient populations [7,8]. Gender differences have also emerged in prescribing studies, with some evidence suggesting female physicians demonstrate more cautious prescribing patterns [9], though the mechanisms underlying these differences remain unclear. Additionally, specialty training influences prescribing attitudes, with Family Medicine physicians potentially developing distinct perspectives due to longitudinal patient relationships and frequent chronic pain management responsibilities [5,10-12]. These observations informed our three primary hypotheses examining rural-urban, gender, and specialty differences in prescribing attitudes.

The awareness-uncertainty paradox

A critical but underexplored question is whether increased awareness of opioid-related risks translates into clearer clinical decision-making or instead generates greater uncertainty. Previous studies have documented significant hesitancy among physicians regarding opioid prescribing practices, with many struggling to balance effective pain management against potential for misuse [13]. This tension is often exacerbated by time constraints, insufficient access to alternative treatments, and inadequate specialist support [14,15]. Notably, existing research indicates that the relationship between risk awareness and clinical confidence requires further characterization [16,17], as it remains unclear whether heightened awareness of opioid-related problems enhances decision-making clarity or contributes to clinical paralysis.

In opioid prescribing, physicians must simultaneously consider pain relief efficacy, addiction risk, regulatory scrutiny, and patient expectations. We hypothesized that this complexity might manifest as an "awareness-uncertainty paradox": physicians who are most cognizant of opioid-related problems in their patient populations may experience greater difficulty with prescribing decisions rather than enhanced confidence. This investigation builds on existing research highlighting the persistent evidence-practice gap and the complex informational challenges clinicians face when making treatment decisions for chronic pain [11,12]. Understanding this relationship has practical implications for designing educational interventions that not only increase risk awareness but also provide actionable decision support.

Study objectives

Therefore, this exploratory study examined demographic predictors of physician opioid prescribing attitudes in Michigan while investigating whether awareness of opioid misuse correlates with prescribing difficulty. We also examined age as a secondary variable to understand how career stage might moderate demographic effects on prescribing attitudes.

## Materials and methods

Study design and participants

This exploratory pilot study developed and administered a survey using the Qualtrics platform (Qualtrics, Provo, UT, USA) to assess opioid prescribing attitudes among practicing physicians in Michigan. Eligibility criteria included being a practicing physician (MD or DO) in Michigan, regardless of specialty or years of experience. After receiving the Institutional Review Board exemption from Central Michigan University (IRB Number: 2024-693, approved December 12, 2024), the survey was distributed via email to practicing physicians across the state through a convenience sampling approach using publicly available physician directories and professional networks. Of the approximately 200 physicians contacted, 51 completed the survey (response rate: 25.5%). Participation was voluntary and anonymous, with respondents able to discontinue at any time.

Survey instrument

The survey questionnaire was developed specifically for this study by the research team through consultation with clinical experts in pain management and addiction medicine. Prior to full deployment, the instrument underwent informal pilot testing with five physicians to assess clarity and completion time (approximately five to seven minutes). The instrument was designed to assess physician demographics, opioid prescribing attitudes, and condition-specific prescribing preferences relevant to the research objectives. The survey assessed demographic characteristics including practice setting, gender, specialty, and age ranges. Opioid prescribing attitudes were measured using three Likert scale items (1=strongly disagree to 5=strongly agree). The first item asked participants to indicate their agreement with the statement, "I believe that incorrect or inappropriate use of opioids is a problem amongst my patients," shortened to "Opioid Misuse is a Problem" in figures. The second item stated, "Prescribing opioids is difficult to deal with," shortened to "Prescribing Opioids is Difficult" in figures. The third item read, "There are many better alternatives instead of opioids to treat chronic noncancer pain," shortened to "Better Alternatives Exist" in figures. A composite mean opioid attitude score was calculated as the average of these three items, with higher scores indicating more cautious attitudes toward opioid prescribing. Internal consistency of the composite scale was assessed using Cronbach's alpha (α=0.62), which falls in the acceptable range (α>0.60) for exploratory research with brief scales, though indicating modest reliability. Additional questions assessed condition-specific willingness to prescribe opioids for cancer pain, sickle cell disease, headaches, and fibromyalgia. The complete survey questionnaire is provided in the Appendix.

Statistical analysis

Survey data were collected via Qualtrics and all analyses were conducted using R Studio (version 4.3.0; R Foundation for Statistical Computing, Vienna, Austria). Missing data affected less than 5% of responses and were handled through listwise deletion. Descriptive statistics summarized demographic characteristics and attitude scores. Primary analyses tested our three main hypotheses using Welch's t-test for comparing mean opioid attitude scores between practice settings and gender groups, and one-way analysis of variance (ANOVA) for comparing Family Medicine physicians to other specialties. All statistical tests were two-tailed with α=0.05.

Statistical assumptions were assessed through normality testing using the Shapiro-Wilk test, homoscedasticity assessment using the Breusch-Pagan test, and multicollinearity evaluation using variance inflation factors <5. For continuous variables, normality was examined using the Shapiro-Wilk test (P>0.05 indicated normal distribution). When parametric assumptions were met, appropriate parametric tests were employed; otherwise, non-parametric alternatives were used.

Secondary analyses examined age effects and potential age×gender interactions using multiple linear regression models. The relationship between awareness of opioid misuse (s03) and prescribing difficulty (s04) was examined using Pearson correlation analysis. Bootstrap resampling (n=1000) assessed the stability of correlation patterns among attitude items.

Effect sizes were reported using Cohen's d for t-tests and eta squared (η²) for ANOVA. P values were expressed to three decimal places, with values less than 0.001 described as "P<0.001." Confidence intervals were reported at the 95% level throughout. Power analysis indicated that with α=0.05 and power=0.800, the sample size was adequate for detecting medium effect sizes (d=0.5) in primary comparisons.

## Results

Sample characteristics

A total of 51 physicians fully completed the survey. The sample comprised predominantly men (37/51, 72.5%) with female representation as well (14/51, 27.5%). Practice settings were primarily rural (37/51, 72.5%) compared to urban (14/51, 27.5%). Family Medicine physicians comprised the majority of the sample (36/51, 70.6%), with other specialties representing (15/51, 29.4%) (Table 1).

**Table 1:** Sample Demographic Characteristics and Primary Outcome Measures Values are presented as n (%) for categorical variables and mean±SD for continuous scores. Hyphens (-) indicate a category header with no direct numerical value. The Opioid Attitude Score is the composite mean of three Likert-scale items (range=1-5; higher scores=more cautious attitudes toward opioid prescribing).

Characteristic	n (%)	Mean Opioid Attitude Score (SD)
Total Sample	51 (100%)	3.60 (0.66)
Practice Setting		
Rural	37 (72.5%)	3.59 (0.67)
Urban	14 (27.5%)	3.62 (0.65)
Gender		
Male	37 (72.5%)	3.64 (0.61)
Female	14 (27.5%)	3.50 (0.78)
Practice Type		
Family Medicine	36 (70.6%)	3.64 (0.67)
Other Specialties	15 (29.4%)	3.51 (0.64)

Condition-specific prescribing endorsement

**Figure 1:**
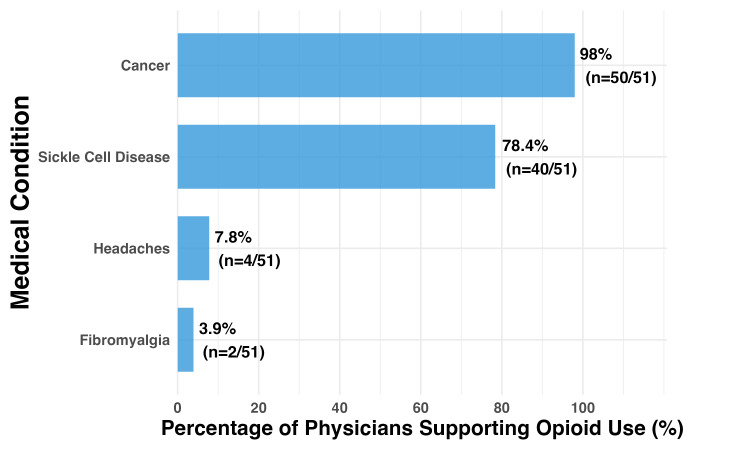
Bar Chart Showing Physician Endorsement of Opioid Therapy by Condition Condition-specific willingness to prescribe opioids among Michigan physicians (N=51). Physicians demonstrated clear hierarchical preferences with near-universal support for cancer pain (50/51, 98.0%) and strong support for sickle cell disease (40/51, 78.4%), while endorsement dropped dramatically for chronic non-cancer conditions including headaches (4/51, 7.8%) and fibromyalgia (2/51, 3.9%). This hierarchy suggests physicians feel most confident prescribing opioids when clear evidence and guidelines exist.

Physicians demonstrated clear hierarchical preferences for opioid therapy across conditions. Nearly universal support existed for cancer pain (50/51, 98.0%) and strong support for sickle cell disease (40/51, 78.4%). However, endorsement dropped dramatically for chronic non-cancer conditions: headaches (4/51, 7.8%) and fibromyalgia (2/51, 3.9%) (Figure 1).

Effect of setting, gender, and practice type

Hypothesis 1 (Rural versus Urban Differences): Contrary to our prediction, no significant differences emerged between rural and urban physicians in opioid prescribing attitudes. Rural physicians (M=3.59±0.67) and urban physicians (M=3.62±0.65) demonstrated statistically indistinguishable attitudes (t(23.99)=0.12, P=0.906, 95% CI (-0.45, 0.4), Cohen's d=0.04).

Hypothesis 2 (Gender Differences): Our hypothesis regarding gender differences was not supported as a main effect. Male physicians (M=3.64±0.61) and female physicians (M=3.50±0.78) showed no significant difference in mean opioid attitude scores (t(19.32)=0.60, P=0.554, 95% CI (-0.62, 0.33), Cohen's d=0.21).

Hypothesis 3 (Practice Type Differences): Family Medicine physicians (M=3.64±0.67) and other specialists (M=3.51±0.64) demonstrated no statistically significant difference in attitudes (F(1,49)=0.396, P=0.532, 95% CI (-0.27, 0.53), η²=0.008).

These results show the effect of setting, gender, and practice type (Table 2).

**Table 2:** Primary Hypothesis Testing Results with 95% Confidence Intervals All tests used α=0.05. CI=confidence interval. Effect sizes: Cohen's d for t-tests (small=0.2, medium=0.5, large=0.8); η² for ANOVA (small=0.01, medium=0.06, large=0.14). Statistical significance is denoted by asterisks (*p<0.05, **p<0.01, ***p<0.001).

Hypothesis	Statistical Test	Test Statistic	P-value	95% CI	Effect Size	Result
Rural vs. Urban	Welch's t-test	t(24) = 0.12	0.906	[-0.45, 0.4]	d = 0.04	Not Supported
Male vs. Female	Welch's t-test	t(19.3) = 0.6	0.554	[-0.62, 0.34]	d = 0.21	Not Supported
Family Med vs. Other	One-way ANOVA	F(1,49) = 0.396	0.532	[-0.27, 0.53]	η² = 0.008	Not Supported

Age×gender interaction effect

Age alone was not a significant predictor of prescribing attitudes (β=-0.25, p=.55), and gender alone was also nonsignificant (β=-0.77, p=.096). However, a statistically significant Age×Gender interaction was observed (β=0.33±0.15, p=.033, 95% CI (0.035, 0.628)), suggesting a potential pattern in which the relationship between age and opioid-prescribing attitudes differs by gender. Given the pilot nature and limited sample size, this finding should be interpreted cautiously and confirmed in larger studies.

**Figure 2:**
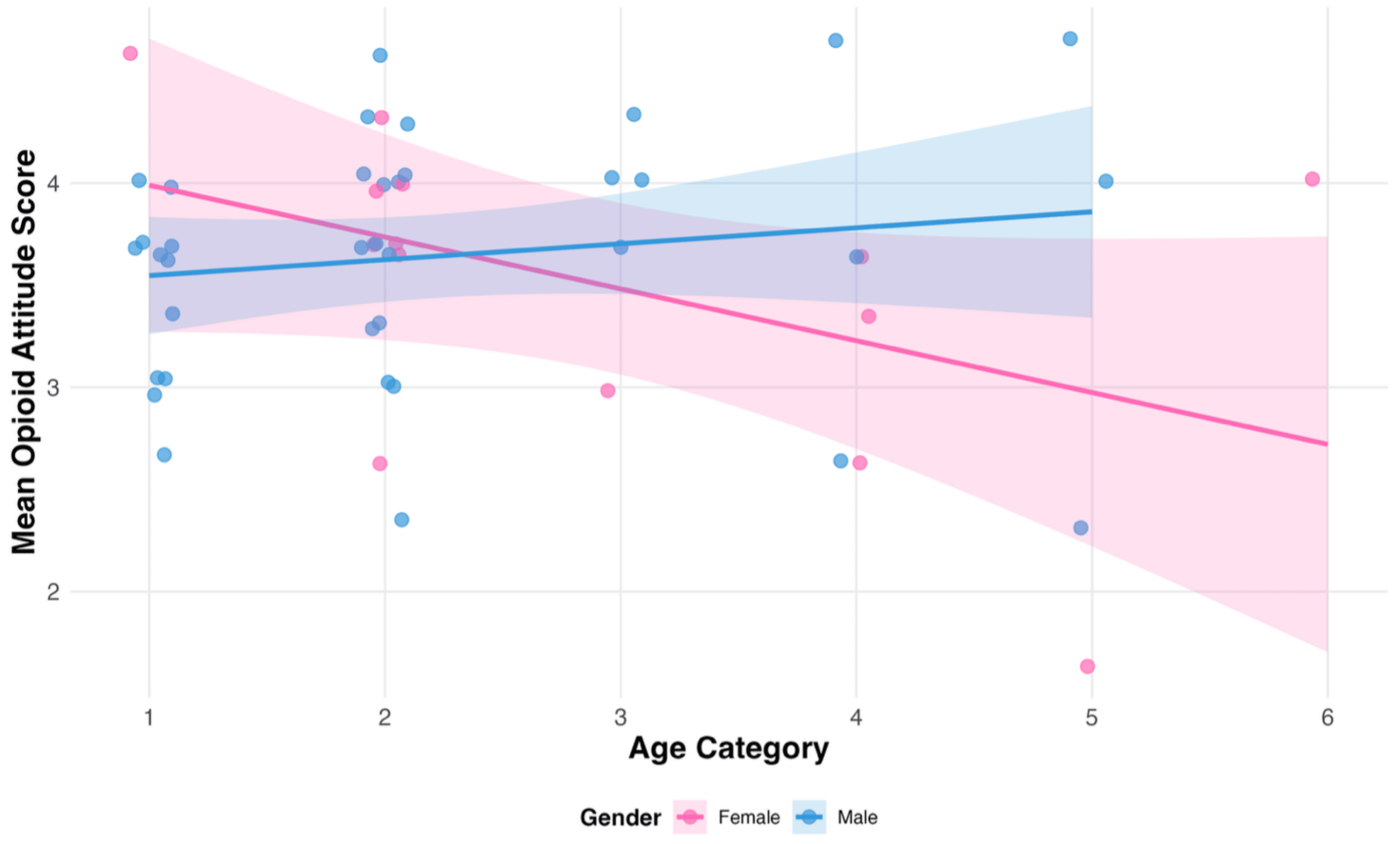
Scatterplot Showing Age × Gender Interaction Effect on Opioid Prescribing Attitudes Interaction between age and gender on mean opioid attitude scores among Michigan physicians (N=51). The significant interaction (β=0.33±0.15, t=2.193, P=0.033) indicates that the relationship between age and prescribing attitudes differs significantly between male and female physicians. Female physicians show decreasing caution with age (pink line, negative slope), while male physicians show increasing caution with age (blue line, positive slope). Higher scores indicate more cautious attitudes toward opioid prescribing. Shaded areas represent 95% confidence intervals. To improve visual clarity, overlapping data points were jittered slightly along the x-axis.

Our findings suggest that gender differences in prescribing attitudes may be moderated by career stage or generational factors, with female physicians showing decreasing caution with age while male physicians show increasing caution with age (Figure 2, Table 3).

**Table 3:** Age × Gender Interaction Analysis Multiple linear regression model: Opioid Attitude Score~ Age+Gender+Age×Gender. R²=0.15, F(3,47)=2.76, P=0.05. Age coded as ordinal variable (1-6 categories), Gender coded as Male=1, Female=0. Statistical significance is denoted by asterisks (*p<0.05, **p<0.01, ***p<0.001). CI: Confidence interval.

Effect	Coefficient (β)	Standard Error	T value	P-value	95% CI	Interpretation
Age	-0.254	0.144	-0.61	0.55	[-0.35, 0.19]	Non-significant main effect
Gender (Male)	-0.774	0.456	-1.698	0.096	[-1.668, 0.119]	Non-significant main effect
Age × Gender	0.330	0.150	2.193	0.033*	[0.035, 0.628]	Age effects differ by gender

Awareness-uncertainty correlation

A key finding emerged from correlation analysis examining the relationship between recognizing opioid misuse as a patient problem (s03) and experiencing prescribing difficulty (s04). These variables demonstrated a strong positive correlation (r=0.611, P<0.001, 95% CI (0.41, 0.76)), indicating that physicians who most recognize opioid-related problems in their patient populations simultaneously experience the greatest difficulty in prescribing decisions.

**Figure 3:**
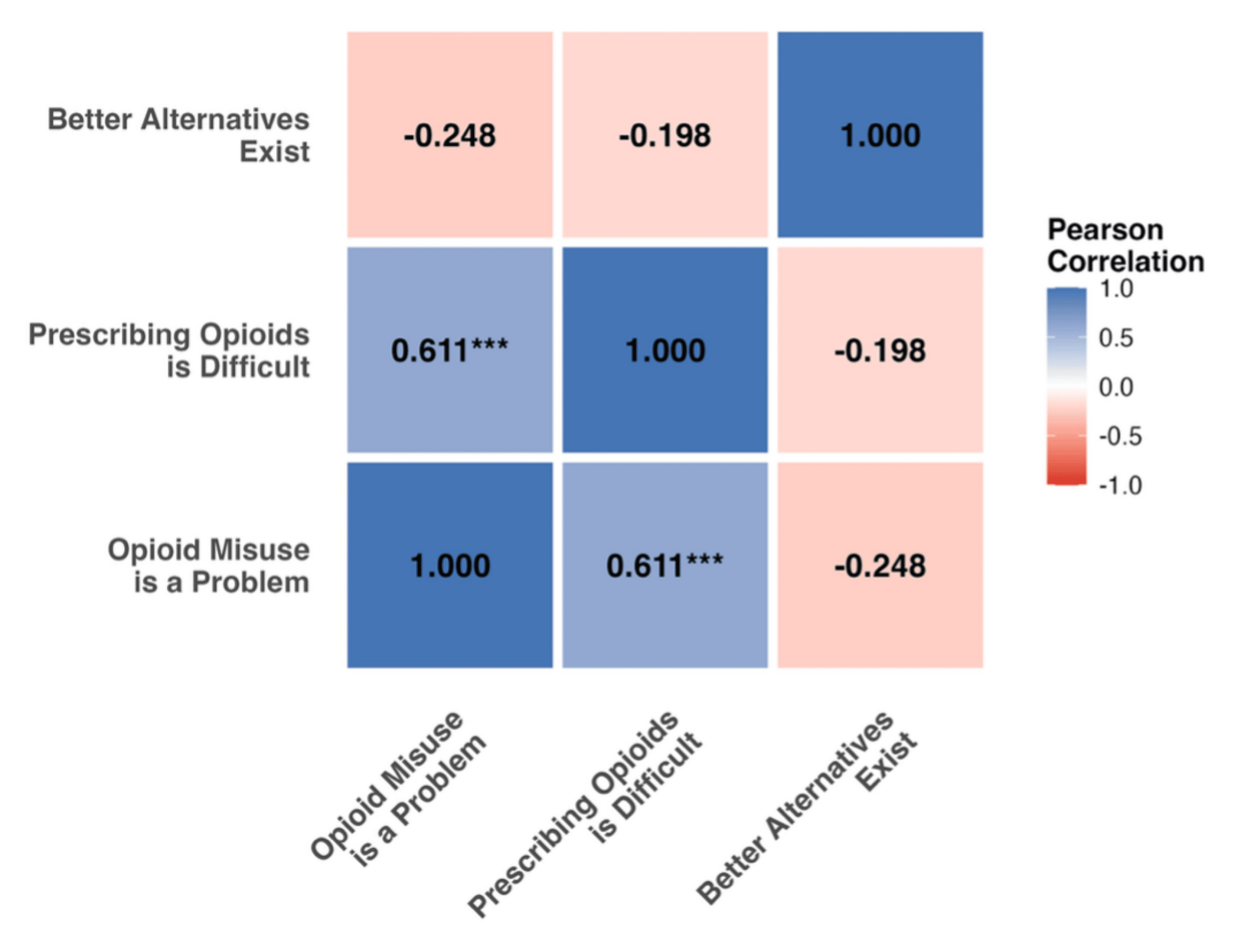
Correlation Matrix of Core Opioid Prescribing Attitude Items Pearson correlation matrix showing relationships between core attitude items among Michigan physicians (N=51). The matrix reveals a strong positive correlation (r=0.611, P<0.001) between recognizing opioid misuse as a patient problem and experiencing prescribing difficulty, suggesting an "awareness uncertainty paradox," where increased risk awareness is associated with greater clinical uncertainty rather than clearer decision making. Color intensity indicates correlation strength (blue=positive, red=negative). Statistical significance is denoted by asterisks (*p<0.05, **p<0.01, ***p<0.001).

Comprehensive correlation analysis of core attitude items revealed that the s03-s04 relationship (r=0.611, P<0.001) was markedly stronger than other correlations: s03-s05 (r=-0.248, P=0.080) and s04-s05 (r=-0.198, P=0.163). This finding suggests that awareness of opioid risks does not simplify clinical decision-making but rather may contribute to increased uncertainty and complexity in practice (Figure 3, Table 4).

**Table 4:** Core Attitude Item Correlations Pearson correlations with 95% confidence intervals. Bootstrap resampling (n=1000) confirmed stability of correlation patterns. Effect sizes: small (r=0.1), medium (r=0.3), large (r=0.5). Statistical significance is denoted by asterisks (*p<0.05, **p<0.01, ***p<0.001).

Comparison	Items	Correlation	P-value	95% CI	Significance	Effect Size
s03 ↔ s04	Opioid Misuse is a Problem ↔ Prescribing Opioids is Difficult	0.611	< 0.001***	[0.41, 0.76]	Highly Significant	Large
s03 ↔ s05	Opioid Misuse is a Problem ↔ Better Alternatives Exist	-0.248	0.08	[-0.49, 0.03]	Not Significant	Small
s04 ↔ s05	Prescribing Opioids is Difficult ↔ Better Alternatives Exist	-0.198	0.163	[-0.45, 0.08]	Not Significant	Small

## Discussion

This exploratory study examined three hypotheses regarding demographic predictors of physician opioid prescribing attitudes in Michigan and uncovered preliminary insights into the relationship between risk awareness and clinical uncertainty. Our findings suggest that simple demographic categorizations may not adequately capture the complexity of prescribing attitudes, while increased awareness of opioid-related risks appeared to be associated with greater prescribing difficulty.

Primary hypothesis findings

Contrary to prior literature suggesting differences in healthcare delivery patterns [7,8,18-23], we found no significant main effects for rural versus urban practice settings, gender, or practice type. Our findings indicated that simple demographic categories did not predict prescribing attitudes, reflecting previous research suggesting that factors such as clinical competence and training background may be more impactful [6,19]. This may convey the pervasive nature of opioid-related concerns across both Michigan and the United States, regardless of geographic location or practitioner characteristics. It may also indicate that statewide policy initiatives and educational efforts have created more uniform prescribing attitudes. This is supported by a study that found a 41.8% decrease in postoperative opioid prescription size between 2013 and 2021; this decline was largely attributed to increasing awareness of opioid risks and efforts to establish safer prescribing practices [24].

Age×gender interaction

The discovery of a significant age×gender interaction in this exploratory pilot study suggests that the relationship between demographics and prescribing attitudes may be more nuanced than main effects would suggest. This preliminary finding suggests that gender differences in prescribing attitudes may be moderated by career stage, with our data indicating that female physicians showed decreasing caution with age, while male physicians showed increasing caution with age in this sample.

This finding provides important context for interpreting previous research regarding gender differences in opioid prescribing. A prior study found that male emergency medicine providers consistently prescribed more opioid tablets than female providers across all scenarios, both when concerned and not concerned about potential misuse [9]. Similarly, another study found that the relationship between physician competence and opioid prescribing varied by gender, with male physicians demonstrating riskier prescribing patterns at lower competence levels compared to female physicians, who showed more cautious practices even with similar competence scores [6]. However, these studies did not examine age interactions, focusing on years of experience as a separate variable. Other studies have also demonstrated that career stage or training level factor into prescribing attitudes; one study highlighted that residents and early-career clinicians often issue larger or less guideline-concordant opioid prescriptions than attendings, potentially indicating that prescribing behavior evolves with experience and stage of practice [20,21].

Our exploratory findings suggest that previously observed gender differences [9] may be more complex than simple categorical differences and may potentially vary based on physician age, career stage, or even competence levels [18-21]. If confirmed in larger samples, the specific interaction pattern we observed could provide a framework for future educational interventions to potentially see benefit in targeting prescribing attitudes with consideration of how age and gender combinations may influence physician responses, rather than treating gender as a uniform predictor across all physician demographics.

The awareness-uncertainty paradox

The strong association between recognizing opioid misuse problems and experiencing prescribing difficulty (r=0.611) represents our most notable finding. This relationship highlights a potential tension in pain management: awareness of opioid-related problems may not simplify prescribing decisions but instead contribute to increased uncertainty and complexity in clinical practice. This aligns with qualitative research suggesting that opioid prescribing behaviors are shaped by complex and often conflicting factors [5], which may help explain why increased awareness of risks does not necessarily translate to clearer decision-making. The relationship between awareness and prescribing difficulty may also reflect broader patterns of physician hesitancy and stigma surrounding opioid prescribing [13]. One study reported that primary care physicians often expressed negative views of patients with opioid use disorder, perceiving them as difficult or manipulative, which contributed to reluctance in prescribing [22]. Barriers to guideline-concordant management, such as limited time and inadequate access to non-opioid alternatives, have also been described and may intensify this uncertainty [23]. In addition, surveys have shown that many providers report discomfort with opioid prescribing despite adequate knowledge, suggesting that awareness without sufficient decision support can heighten uncertainty [16]. Therefore, educational interventions may need to address not only knowledge gaps but also confidence and comfort with prescribing decisions in complex clinical scenarios.

This finding challenges common assumptions about the relationship between education and clinical confidence. Rather than providing clarity, recognition of opioid risks appeared to create additional challenges for practitioners navigating the balance between adequate pain management and safety concerns. The clinical implications of this observation warrant further investigation. Current educational approaches that focus primarily on risk identification may inadvertently contribute to clinical uncertainty if not paired with appropriate decision-making mechanisms. To reduce uncertainty, future educational interventions might benefit from coupling risk awareness with practical frameworks and tools that equip physicians to navigate complex clinical scenarios with confidence.

Clinical decision-making hierarchy

The condition-specific hierarchy we observed, with near-universal support for cancer pain (n=50/51, 98.0%) but minimal endorsement of chronic non-cancer conditions such as fibromyalgia (n=2/51, 3.9%), provides insight into how physicians have developed intuitive clinical guidelines. This hierarchy suggests that physicians feel confident prescribing opioids when clear evidence and guidelines exist (cancer pain) but experience significant uncertainty in areas where evidence is more complex or controversial (chronic non-cancer pain). This is corroborated in a similar study done in the state of Wisconsin surveying the attitudes and beliefs of physicians towards opioid prescription. The study found that most providers were willing to prescribe opioids for chronic cancer pain, but only about half were willing to do the same for non-cancer pain [25]. This pattern reinforces the importance of developing clear, evidence-based guidelines for chronic pain management [11,12] and providing physicians with practical tools for implementing these guidelines in complex clinical scenarios.

Implications for medical education and clinical practice

These preliminary findings have potential implications for educational interventions and clinical support systems. The absence of simple demographic main effects suggests that one-size-fits-all approaches to continuing medical education may be insufficient. The observed age×gender interaction indicates that educational programs may need to consider how different demographic combinations of physicians respond to educational interventions. Previous research has documented challenges with provider confidence in opioid prescribing [16], and our preliminary findings suggest that increased risk awareness may paradoxically reduce confidence rather than enhance decision-making clarity.

The awareness-uncertainty association observed in this study highlights the potential value of decision-support tools and practical frameworks that guide physician prescribing. Educational efforts may be strengthened by pairing risk awareness with clear strategies for decision-making, such as the development of structured decision-making frameworks, risk stratification tools, and communication techniques that allow for effective pain management and safe opioid use.

Study limitations and future directions

Our study has several limitations that suggest important directions for future research. The most significant limitation is our small sample size (n=51), which restricts statistical power and limits generalizability, particularly for interaction analyses. This exploratory cross-sectional design provides a snapshot of attitudes but cannot address how these attitudes may evolve over time or in response to policy changes. Our use of convenience sampling through physician directories and professional networks introduces potential selection bias, as physicians who chose to respond may differ systematically from non-responders in their opioid prescribing attitudes, risk awareness, or clinical confidence. Our exclusive focus on Michigan physicians restricts generalizability, as attitudes may differ in states with different regulatory environments, patient populations, or practice cultures. Additionally, self-reported survey data may be subject to response bias, particularly given the sensitive nature of opioid prescribing in the current regulatory environment. The internal consistency of our attitude scale (Cronbach's α=0.62) was adequate but modest, and future research might benefit from validated instruments such as the Clinicians' Attitudes and Beliefs about Opioids Survey to capture more nuanced attitudinal dimensions. To address these limitations, future research should examine the age× gender interaction in larger, more diverse samples to confirm these preliminary patterns and explore their underlying mechanisms. Investigating the link between actual prescribing behaviors and patient outcomes would provide important confirmation of these findings and reveal the clinical significance of observed attitude patterns. Longitudinal studies could clarify how attitudes evolve over time and in response to policy changes or educational programs. Qualitative research using in-depth interviews or focus groups exploring the mechanisms behind the awareness-uncertainty association and age×gender interaction would be particularly valuable for developing targeted interventions and could provide richer insights into physician decision-making processes than survey data alone. Multi-state studies would be valuable to determine whether the patterns observed in Michigan generalize to other geographic contexts with different regulatory environments, patient demographics, and healthcare delivery systems. Finally, investigation of decision-support tools that specifically address the tension between risk awareness and prescribing uncertainty could yield practical solutions to the challenges identified in this study.

## Conclusions

This exploratory pilot study provides preliminary evidence suggesting several areas that may be useful for future research. We found no clear differences in prescribing attitudes based on practice setting, gender, or specialty in this sample. However, the age x gender interaction suggests the possibility that these factors may influence prescribing in more complex ways than single categories can reveal, though this finding requires validation in larger, more representative samples. The link between recognizing opioid misuse as a patient issue and experiencing greater prescribing difficulty raises important questions about whether increased risk awareness can increase clinical prescribing uncertainty rather than reduce it. The stronger support for opioid use in conditions like cancer and sickle cell disease, compared to limited support for chronic non-cancer conditions, suggests that physicians may feel more confident when evidence and guidelines are clearer. These preliminary findings could help guide future studies and inform the development of training and resources that better address the realities of prescribing in uncertain or high-risk situations. Future research with larger, more diverse samples is essential to validate these exploratory associations and determine their generalizability beyond Michigan. As Michigan continues to address its opioid crisis, recognizing the complexity of physician attitudes and decision-making processes may be valuable for developing targeted interventions that promote both effective pain management outcomes and opioid safety.

## Appendices

**Table 5:** Complete Survey Questionnaire This table reproduces the full survey instrument administered to practicing physicians in Michigan. It lists each questionnaire section, question number (Q#), verbatim question text, and available response options. Opioid prescribing attitudes were assessed with three Likert-scale items (Items 6-8: “Opioid misuse is a problem,” “Prescribing opioids is difficult,” “Better alternatives exist”); the Composite Opioid Attitude Score was calculated as the mean of these three items (range = 1-5; higher scores indicate more cautious attitudes). Variable coding used in analyses: Practice Setting=Rural 0, Urban 1; Gender=Female 0, Male 1; Specialty=Other 0, Family Medicine 1; Age coded ordinally (1-6) to match listed ranges. The survey was built and distributed through Qualtrics, completed anonymously in approximately five to seven minutes, and approved by the Central Michigan University Institutional Review Board.

Physician Opioid Prescribing Attitudes Survey			
Section	Q#	Question Text	Response Options
Consent		Statement of consent	By continuing with this survey, I am indicating that I am 18 years of age or older and that I consent to participate in this research.
Demographics	1	What is your age?	25-35 years / 36-45 years / 46-55 years / 56-65 years / 66-75 years / 76+ years
	2	What is your gender?	Male / Female / Non-binary / Prefer not to answer
	3	What is your practice setting?	Rural / Urban
	4	What is your medical specialty?	Family Medicine / Internal Medicine / Emergency Medicine / Anesthesiology / Surgery / Psychiatry / Other (please specify)
	5	How many years have you been practicing?	0-5 years / 6-10 years / 11-15 years / 16-20 years / 21+ years
Opioid Prescribing Attitudes	6	I believe that incorrect or inappropriate use of opioids is a problem amongst my patients.	Strongly disagree / Disagree / Neither agree nor disagree / Agree / Strongly agree
	7	Prescribing opioids is difficult to deal with.	Strongly disagree / Disagree / Neither agree nor disagree / Agree / Strongly agree
	8	There are many better alternatives instead of opioids to treat chronic noncancer pain.	Strongly disagree / Disagree / Neither agree nor disagree / Agree / Strongly agree
Condition-Specific Prescribing	9	For which of the following conditions would you consider prescribing opioids? (Check all that apply)	Cancer pain / Sickle cell disease / Headaches / Fibromyalgia
